# Appendicitis: A Brief Review of My Personal Experience

**Published:** 1895-07

**Authors:** Floyd Wilcox McRae

**Affiliations:** Atlanta


### Southern Medical Record.

A MONTHLY JOURNAL OF MEDICINE AND SURGERY.

VOL. XXV. ATLANTA, GA., JULY, 1895. No. 7.

### Original Articles.

### APPENDICITIS: A BRIEF REVIEW OF MY PERSONAL EXPERIENCE.

By FLOYD WILCOX McRAE, Atlanta.

Appendicitis, though much written about and frequently discussed, is a subject of perennial interest to both surgeon and general practitioner; and there is such a great diversity of opinion as to the proper management of appendicitis, that frequent discussion is necessary for a proper solution of the vexatious questions which present themselves to us in every instance. It is only by constantly comparing notes, both of the strictly medical cases, and those which come to operation, and carefully compiling statistics of non-operative as well as operative cases, that we can ever hope to arrive at the truth. I am firmly of the opinion that many cases of appendicitis are strictly medical cases, but I think every case should be considered from the onset as probably surgical. In no disease is it more important for physician and surgeon to work together harmoniously, each lending to the other the value of his experience and best judgment, without dogmatism and without jealousy. In this paper I shall confine my remarks to the diagnosis and treatment of appendicitis.

In every case where there is a sudden attack of abdominal pain, especially if there be nausea and fever, appendicitis should be suspected and carefully sought for. In many cases it can be excluded, but it is always important to bear in mind the possibility of appendicitis and make a diagnosis at the earliest possible moment.

Even in the beginning there are signs which point to appendicitis with more or less certainty. One of the first signs that may be observed is that of limited movement of the abdominal muscles of the right side during respiration. The four cardinal symptoms, as laid down by Murphy, "sudden attack of pain over the appendix; always nausea, frequently vomiting; elevation of temperature; exaggerated local tenderness in various positions occupied by the appendix," should always be borne in mind. Occasionally the pain and tenderness become localized on the opposite side of the abdomen or in the epigastric region. In some cases it is referred especially to the bladder, and the only symptoms complained of are pains in the bladder, and either difficulty in emptying the bladder or frequent micturition. In two of the cases which I have operated upon this symptom has been especially well marked. After a tumor has developed it may usually be detected by palpation through the abdominal walls. Examination per rectum is often of value in clearing up the diagnosis, and in the female, vaginal examination is of great value. The rapidity with which the tumor develops varies much in different cases, sometimes developing with great rapidity, and in other instances only becoming apparent after a number of days. The rigidity of the abdominal muscles on the right side, and especially of the right rectus abdominalis, is present almost without exception, and is of great value in directing our attention to the probability of this disease. Pain is of not as great value in the diagnosis as localized tenderness in the situations usually occupied by the appendix. Where the tenderness constantly increases, spreading over a larger and larger area, it is evidence that the disease is steadily advancing. When, however, the pain grows less, the tenderness becoming more and more circumscribed, the temperature falls and the pulse shows a marked improvement in quality, it is probable that resolution, more or less complete, will follow. In typical cases of appendicitis the diagnosis should be as readily made as in either pleurisy or pneumonia.

In the atypical cases of appendicitis a positive diagnosis is often difficult, sometimes impossible, prior to opening the abdominal cavity. Except in the fulminant cases the treatment should be medical in the beginning; the bowels should be thoroughly purged, the diet regulated, counter irritation or cold applied over the seat of the pain and tenderness, and the progress of the attack watched carefully. Where the symptoms do not subside after careful purging, and local treatment indicated, the case should be considered from a surgical standpoint, and where the pain and tenderness are increasing, the general symptoms becoming more and more marked, an operation should be done without delay, where favorable conditions and a good surgeon are at hand. I would, however, very much rather risk my chances if I had appendicitis, in the hands of a good physician, than of an unclean surgeon who has more daring than skill or judgment.

When an operation has been determined upon every precaution should be taken to make it thoroughly aseptic. Too great care cannot be given to the cleansing of the hands of the surgeon and his assistant, the field of operation, and the preparation of instruments and dressings. These details should be none the less carefully carried out where there is a general septic peritonitis or a localized abscess. A surgeon is inexcusable who adds to the already infected tissues new infection. Where there is a distinct localized abscess, with well-defined walls shutting it off from the general peritoneal cavity, no attempt should be made to remove the appendix, unless it appears in the field of operation or may be removed without breaking up the protective adhesions. It is a rule that an appendix which has once suppurated freely, forming a localized abscess, or terminated in general peritonitis, does not again become diseased. In these conditions the surgeon should attempt to save life and not to do ideal surgery. Should the appendix subsequently give trouble it may be removed when the conditions are more favorable. Here the surgeon should satisfy himself with a careful cleansing of the abscess cavity, thorough packing with gauze; and glass or rubber drainage, where there is alocalized abscess; with a careful toilet and thorough cleansing of the peritoneal cavity, and free drainage where there is a general peritonitis.

Since our last meeting I have seen in my own practice and in consultation nine patients suffering with appendicitis, and two of the nine patients having had each two attacks. I have also seen one patient referred to me by his physician, who had treated him through several attacks of recurrent appendicitis, in the interval between the attacks. Of these ten patients, I have operated on four, and assisted Dr. Nicolson in one, making five cases that have come to operation. Of the five patients not operated upon, one was a very old woman, a morphine habitue, who died of the general peritonitis and exhaustion a short time after I first saw her. The other four patients, two of whom each had two attacks, have been under my observation, and are now apparently in good health. Of the five cases operated upon, three of mine and one operated upon by Dr. Nicolson, recovered. One of the cases operated upon by myself was an old man, who died nine weeks after the operation from exhaustion—paresis of the bowels.

I append below brief histories of my operative cases.

CASE I.—Julia H., aged twenty-two, single. No history of previous attacks. First called to see her Sunday, February 25th. Found her complaining of pain and tenderness in right iliac region, temperature 101°, pulse 80. Prescribed salts in teaspoonful doses until bowels were freely purged. On Tuesday she came to my office and was feeling much better. I did not see her again until following Sunday, when I found her suffering very much as she was at my first visit one week before, and prescribed the same treatment. Monday she was no better, Tuesday she was much worse, and Wednesday her condition was extremely critical. Temperature 102°; pulse 140 to 150; abdomen immensely distended. Operation March 7th, incision, drainage, recovery. (Previously reported in a paper read before the Medical Association of Georgia.)

CASE II.—Jacob B., aged twenty-eight, lawyer. History of repeated attacks of colic, and one or more of inflammation of bowels. First saw him in consultation with attending physician, May 10th or 11th. Found him suffering with pain and tenderness in right iliac region, irregular temperature, ranging from 99° to 103°. His physician had diagnosed the case typhoid fever, and I was called to help determine whether he could go to his home fourteen miles in the country or not. I was not sure what his trouble was, but rather inclined to the idea that it was appendicitis. He was carried to his home, and I heard no more from him until the night of May 15th, when I was called to operate on him for rupture of the appendix and general peritonitis.

Operation May 16th. Ether. Lateral incision; assisted by Drs. J. L. and A. A. Barge. Found appendix completely bound down by old and recent adhesions, slightly distended with very foul pus, beginning general peritonitis. Removed appendix, washed out abdominal cavity with hot water, inserted rubber and gauze drains, closed wound with silkworm gut sutures, and dressed in usual way. Strychnia and hot water bottles were used freely to bring about reaction. Bowels were kept open with salts. Case progressed very favorably until May 26th, when he was seized with violent cramps in bowels, requiring the hypodermic injection of morphine to relieve it. From an oversight the bowels had not been moved for four or five days. Vomiting set in, and was so violent that everything taken into the stomach was rejected almost immediately. Impossible to move bowels.

Second operation May 28th. Assisted by Drs. J. L. and A. A. Barge, Kennedy and Hubbard. Chloroform. Patient *in extremis*. Reopened former incision with finger. Found complete paresis of bowels, adhesion of omentum to cæcum, and of several coils of small intestines to cæcum and to each other. Broke up adhesions, packed wound with gauze and dressed in usual way. Vomiting continued. Gave strychnia, salts and elaterin hypodermically, and succeeded in getting slight movement of bowels on May 31st, followed by free evacuations. His improvement had been steady, and he was convalescent on June 13th, when I last heard from him. Recovery complete and uninterrupted.

CASE III.—Walter W., aged sixteen, scrofulous diathesis. History of repeated attacks of stomachache, sometimes accompanied with fever. First called Sunday, June 17th. Found him with temperature of 101°, pulse 84, nauseated and vomiting occasionally, pain in epigastrium. Prescribed saccharated calomel, to be followed by saline. Called again next morning, found temperature 103°, pulse 96, pain and tenderness over the site of the appendix very marked, muscles very tense, slight induration and dullness. Diagnosed appendicitis. Told family I thought an operation would be essential if he was not improved by next morning. Called Dr. Nicolson in consultation, and he agreed with diagnosis, and we put patient on teaspoonful doses of salts in hot water, to be repeated hourly until bowels were freely purged. Next morning, Tuesday, June 19th, temperature 103°, pulse 108. Operated at noon, assisted by Drs. Nicolson, Kennedy and Hubbard. Lateral incision. Found appendix very much enlarged, gangrenous, and contained in an exceedingly thin-walled abscess, holding about four ounces of very dark, thin, fetid pus, which must have ruptured into general peritoneal cavity in a few hours. Removed appendix, inserted gauze and rubber drains, closed and dressed in usual way.

Tedious recovery. He developed a fecal fistula on fourth day, which discharged freely for about three weeks, but closed spontaneously. Developed an abscess low down in pelvis, fourth week, which was evacuated through original incision, by tearing up adhesions with the finger. Ligature with which the appendix was tied off, came away on thirty-sixth day. Recovery.

A ventral hernia developed several months after the operation. The boy was so averse to any kind of constraint that he would not wear either adhesive strips, or any abdominal supporter. The hernia is small, and has never given him any inconvenience, but will probably grow worse, unless carefully supported.

CASE IV.—Thomas J., aged about forty. Seen in consultation, September 25th, with Dr. L. P. Stevens. History of sudden attack of colic, followed by jaundice, three weeks previous to my first visit. The attack came on soon after the patient had eaten heartily of watermelon. The patient vomited freely. Suffered with violent colicky pains for several hours, but did not consult his physician until about a week afterwards, and then simply asked him for a prescription for biliousness. Dr. Stevens, when first consulted, suspected appendicitis, and sent his patient home, and made an examination that night, but could find no well-defined tumor. Temperature ranged from 99° to 101°, and rarely above 99½°. Pulse, about 70 to 80. Pain in the bladder was complained of continually. When I first saw him, there was a well-defined tumor in the right iliac fossa. Marked tenderness, temperature 99½°, pulse 72. Operation was advised, and upon the following day I operated—September 26—at patient's home, assisted by Drs. L. P. Stevens, J. P. Kennedy, T. V. Hubbard, and Medical Student C. E. Boynton. Lateral incision. Well-defined abscess (just on the point of rupture, into general cavity) completely walled off by dense adhesions, except point of imminent rupture, which immediately came into view. The slightest manipulation ruptured the abscess wall, but fortunately the general peritoneal cavity was protected by careful sponging. This abscess contained about four ounces of pus. The tumor, including the appendix, was removed entire. Subsequent history uneventful; recovery prompt and complete; two or three of the ligatures used in tying off the omentum, and the one with which the appendix was tied off, subsequently came away through a small fistulous opening in the upper angle of the wound.

CASE V.—Newt. McL., aged about fifty-five, resident of Andrews, N. C. Seen in consultation with Dr. Donald Wilson, February 22d. History of one attack of bilious colic, with fever and vomiting, about a year ago. Symptoms at first very indefinite. Pain in the bladder was so marked, and other symptoms were so obscure, that it was about two weeks before a positive diagnosis was made, and nearly three weeks before the operation was done. When I first saw him, there was a large tumor occupying the right iliac fossa, and extending as far as the median line. Fluctuation easily detected. Temperature, 101°; pulse, 112. General condition, fairly good, though patient was extremely despondent. Operation immediately determined upon, and preparation rapidly made.

Operation, February 22d. Chloroform. Assisted by Drs. Wilson and Webb, of Andrews, N. C., and Dr. Greenwood, of Mineral Bluff, Ga. Incision made into the large localized abscess, containing more than a pint of very foul pus. The abscess cavity was thoroughly cleansed with sponging, and washed with sterilized water and peroxide of hydrogen. Packed with gauze, and a large rubber draining tube inserted. The temperature rapidly fell to 97½°, and the pulse to about 84 to 90. General condition of the patient following operation was excellent. I left that night, and did not see the patient again. He improved nicely for the first three or four weeks, the only difficulty being to get him to take sufficient nourishment, and to keep his bowels open. April 5th, the patient died, probably from paresis of the bowels, due to the dense and extensive adhesions which had formed during the slow development of the attack during the three weeks preceding the operation.

